# Epinephrine, vasopressin and steroids for in-hospital cardiac arrest: the right cocktail therapy?

**DOI:** 10.1186/cc13903

**Published:** 2014-06-02

**Authors:** Jaya P Buddineni, Clifton Callaway, David T Huang

**Affiliations:** 1 Department of Critical Care Medicine, University of Pittsburgh, Pittsburgh, PA 15261, USA; 2 Department of Emergency Medicine, University of Pittsburgh, Pittsburgh, PA 15210, USA

## Abstract

### Citation

Mentzelopoulos SD, Malachias S, Chamos C, Konstantopoulos D, Ntaidou T, Papastylianou A, Kolliantzaki I, Theodoridi M, Ischaki H, Makris D, Zakynthinos E, Zintzaras E, Sourlas S, Aloizos S, Zakynthinos SG: **Vasopressin, steroids, and epinephrine and neurologically favorable survival after in-hospital cardiac arrest: a randomized clinical trial.***JAMA* 2013, **310:**270**–**279.

### Background

Among patients with cardiac arrest, preliminary data have shown improved return of spontaneous circulation and survival to hospital discharge with the vasopressin-steroids-epinephrine (VSE) combination.

### Methods

*Objective: *To determine whether combined vasopressin-epinephrine during cardiopulmonary resuscitation (CPR) and corticosteroid supplementation during and after CPR improve survival to hospital discharge with a Cerebral Performance Category (CPC) score of 1 or 2 in vasopressor-requiring, in-hospital cardiac arrest.

*Design:* Randomized, double-blind, placebo-controlled, parallel-group trial performed from 1 September 2008 to 1 October 2010.

*Setting:* Three Greek tertiary care centers.

*Subjects:* Consecutive in-hospital cardiac arrest patients requiring epinephrine and aged more than 18 years (n = 268).

*Intervention:* Patients received either vasopressin (20 IU/CPR cycle) plus epinephrine (1 mg/CPR cycle; cycle duration approximately 3 minutes; VSE group, n = 130) or saline placebo plus epinephrine (1 mg/CPR cycle; cycle duration approximately 3 minutes; control group, n = 138) for the first 5 CPR cycles after randomization, followed by additional epinephrine if needed. During the first CPR cycle after randomization, patients in the VSE group received methylprednisolone (40 mg) and patients in the control group received saline placebo. Shock after resuscitation was treated with stress-dose hydrocortisone (300 mg daily for 7 days maximum and gradual taper; VSE group, n = 76) or saline placebo (control group, n = 73).

*Outcomes:* Return of spontaneous circulation (ROSC) for 20 minutes or longer and survival to hospital discharge with a CPC score of 1 or 2.

### Results

Follow-up was completed in all resuscitated patients. Patients in the VSE group versus patients in the control group had higher probability for ROSC for 20 minutes or longer (109/130 (83.9%) versus 91/138 (65.9%); odds ratio (OR), 2.98; 95% confidence interval (CI), 1.39 to 6.40; *P* = 0.005) and survival to hospital discharge with CPC score of 1 or 2 (18/130 (13.9%) versus 7/138 (5.1%); OR, 3.28; 95% CI, 1.17 to 9.20; *P* = 0.02). In the subset of subjects with post-resuscitation shock, subjects in the VSE group versus patients in the control group had higher probability for survival to hospital discharge with CPC scores of 1 or 2 (16/76 (21.1%) versus 6/73 (8.2%); OR, 3.74; 95% CI, 1.20 to 11.62; *P* = 0.02), improved hemodynamics and central venous oxygen saturation, and less organ dysfunction. Adverse event rates were similar in the two groups.

### Conclusions

Among patients with cardiac arrest requiring vasopressors, combined vasopressin-epinephrine and methylprednisolone during CPR and stress-dose hydrocortisone in postresuscitation shock resulted in improved survival to hospital discharge with favorable neurological status compared with epinephrine.

## Commentary

In-hospital cardiac arrest is associated with significant morbidity and mortality. Among in-hospital post-cardiac arrest patients, survival to hospital discharge is approximately 20%. Among these survivors, the prevalence of severe disability or vegetative state ranges from 25% to 50% [1].

During resuscitation from cardiac arrest, coronary perfusion pressure is driven by the difference between aortic diastolic pressure and right atrial pressure [2,3]. Vasopressor drugs act primarily by increasing aortic diastolic pressure and systemic vascular resistance, thus increasing coronary perfusion pressure. For many years epinephrine has been the standard vasopressor recommended in Advanced Cardiac Life Support algorithms. Epinephrine acts on both α- and β-receptors, increasing peripheral vasoconstriction and cardiac stimulation. The α-adrenergic effects are believed to be primarily responsible for increasing myocardial and cerebral blood flow and facilitating return of spontaneous circulation (ROSC). The β-effects of epinephrine on the heart are not beneficial during or after cardiac arrest, and may worsen post-resuscitation myocardial dysfunction and increase myocardial oxygen consumption [4,5].

Vasopressin, an endogenous peptide synthesized in the hypothalamus, has been proposed as an adjunct to epinephrine in management of cardiac arrest [6]. Vasopressin causes vasoconstriction of skin, skeletal muscle, and splanchnic circulation, thus increasing peripheral arterial resistance [7]. These effects are mediated via V1 receptors, but unlike epinephrine, vasopressin has no direct effects on the myocardium [8]. Vasopressin dilates cerebral blood vessels to a greater extent than epinephrine, it has a longer half-life, and its effect is not diminished by acidosis, common in prolonged cardiac arrest [8]. Further support for possible benefits of vasopressin during cardiac arrest comes from the observation that vasopressin levels in post-cardiac arrest patients are higher in survivors compared to non-survivors [9,10]. The role of steroids in stress states has been well studied and may be beneficial in cardiac arrest patients for several reasons. Steroids attenuate the systemic inflammatory response syndrome (SIRS) and improve cerebral perfusion. Cardiac arrest is a high stress state that is associated with SIRS-like response [6,11], decreased perfusion of the adrenal glands, and lower cortisol levels during and after cardiopulmonary resuscitation (CPR) [6]. The release of adrenal hormone *per se* is impaired in post-cardiac arrest patients, thus leading to inadequate response to the physiological insult [11-13]. The beneficial role of adrenal activation during cardiac arrest is supported by the high adrenocorticotropic hormone levels in successfully resuscitated cardiac arrest patients [14] and low serum cortisol levels in patients with early post-resuscitation mortality [11]. In addition, steroids potentiate the effects of the vasoconstrictors by facilitating intracellular signaling by vasoconstrictor receptors [15,16].

Based on the above rationale, the authors first demonstrated beneficial effects of the combination of epinephrine, vasopressin and corticosteroids for in-hospital cardiac arrest in a small, single-center pilot randomized clinical trial (n = 100) [17]. The authors found that the intervention group when compared to the control group had more frequent ROSC (81% versus 52%), and improved survival to hospital discharge (19% versus 4%). This study, however, lacked neurologic outcome data, and had a small sample size.

The authors therefore performed a multicenter randomized control trial in three tertiary centers in Greece, with a larger sample size (300 subjects), using survival to hospital discharge with good neurologic function (Cerebral Performance Category (CPC) score 1 or 2) as the primary outcome. A single randomization point assigned subjects to a treatment strategy of vasopressin, epinephrine, and corticosteroids (VSE group) or a treatment strategy of only epinephrine (Epi group). In this study, the VSE group showed a higher rate of ROSC, and increased survival to hospital discharge with preserved neurological function compared to the Epi group. The authors concluded that adding vasopressin and steroids to epinephrine during the resuscitation phase of in-hospital cardiac arrest and continuing use of steroids in post-resuscitation shock improves survival and neurological outcomes.

In a disease state with very poor survival rate, this is the first randomized controlled trial showing positive neurological outcomes with pharmacotherapy. The study authors used multiple methods to confirm internal validity. They deployed chromatography to ascertain drug stability, used appropriate double blinding (masked allocation) throughout the course of the study, and employed sound monitoring protocols with high follow-up rates. The authors acknowledged crossover contamination in both arms.

A major limitation of this study is the use of multiple interventions at the same time in both arms, making it difficult to discern which one of these interventions causes benefit. Most of the difference in survival between groups was already apparent at ROSC, raising some question as to whether the subsequent steroids in the post-cardiac arrest care were necessary. Nonetheless, the combination VSE strategy showed improved neurological outcomes in a condition with extremely poor survival. It is possible that each of these drugs alone is insufficient to affect outcome, but the combination is synergistic. Alternatively, the VSE regimen resulted in less total epinephrine use, and the beneficial effect of VSE may be related to reduced harm from high doses of epinephrine. Further trials with multi-arm intervention groups using different combinations of epinephrine, vasopressin and steroids may delineate the precise role of each individual agent.

The authors demonstrated improved outcomes using a combination of epinephrine, vasopressin and high dose steroids. The benefits of this triple-agent combination along with its ease of implementation and low cost offset the risks in a disease with such high mortality, making these treatment options attractive for immediate use in clinical practice.

## Recommendation

Given the paucity of proven interventions for, and the high mortality in, hospital cardiac arrest patients, it is reasonable to consider a combination of vasopressin, steroids and epinephrine during resuscitation and during the post-resuscitation shock phase.

## Abbreviations

CI: Confidence interval; CPC: Cerebral performance category; CPR: Cardiopulmonary resuscitation; OR: Odds ratio; ROSC: Return of spontaneous circulation; SIRS: Systemic inflammatory response syndrome; VSE: Vasopressin-steroids-epinephrine.

## Competing interests

The authors declare that they have no competing interests.
